# Cardiovascular Risk after Kidney Transplantation: Causes and Current Approaches to a Relevant Burden

**DOI:** 10.3390/jpm12081200

**Published:** 2022-07-23

**Authors:** Francesco Reggiani, Gabriella Moroni, Claudio Ponticelli

**Affiliations:** 1 Nephrology and Dialysis Unit, IRCCS Humanitas Research Hospital, Via Manzoni 56, Rozzano, 20089 Milan, Italy; francesco.reggiani@hunimed.eu (F.R.); gabriella.moroni@hunimed.eu (G.M.); 2 Department of Biomedical Sciences, Humanitas University, Pieve Emanuele, 20090 Milan, Italy; 3 Independent Investigator, 20131 Milan, Italy

**Keywords:** kidney transplantation, cardiovascular disease, cardiovascular risk factors

## Abstract

Background. Cardiovascular disease is a frequent complication after kidney transplantation and represents the leading cause of mortality in this population. Material and Methods. We searched for the relevant articles in the National Institutes of Health library of medicine, transplant, cardiologic and nephrological journals. Results. The pathogenesis of cardiovascular disease in kidney transplant is multifactorial. Apart from non-modifiable risk factors, such as age, gender, genetic predisposition and ethnicity, several traditional and non-traditional modifiable risk factors contribute to its development. Traditional factors, such as diabetes, hypertension and dyslipidemia, may be present before and may worsen after transplantation. Immunosuppressants and impaired graft function may strongly influence the exacerbation of these comorbidities. However, in the last years, several studies showed that many other cardiovascular risk factors may be involved in kidney transplantation, including hyperuricemia, inflammation, low klotho and elevated Fibroblast Growth Factor 23 levels, deficient levels of vitamin D, vascular calcifications, anemia and poor physical activity and quality of life. Conclusions. The timely and effective treatment of time-honored and recently discovered modifiable risk factors represent the basis of the prevention of cardiovascular complications in kidney transplantation. Reduction of cardiovascular risk can improve the life expectancy, the quality of life and the allograft function and survival.

## 1. Background

**Figure 1:**
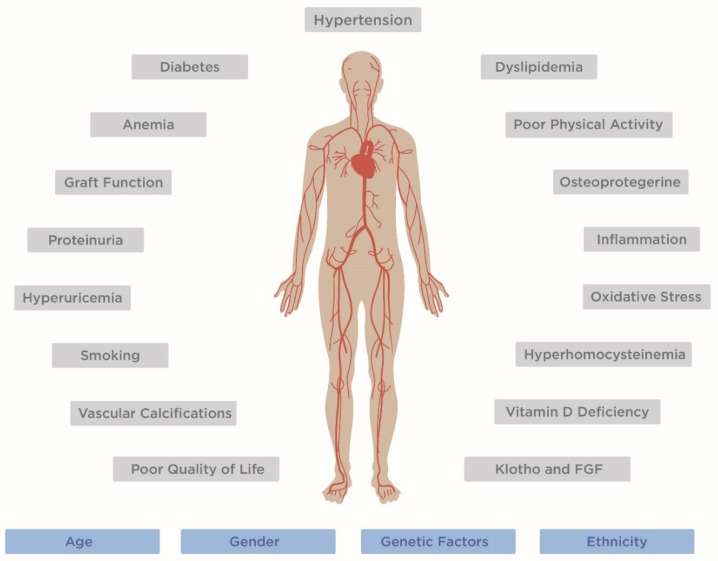
Factors involved in CVD development in kidney transplant recipients. Non-modifiable risk factors are marked in blue, modifiable risk factors are marked in grey. FGF, fibroblast growing factor.

Cardiovascular disease (CVD) is a frequent cause of morbidity after kidney transplantation (KT) and represents the leading cause of mortality [1,2,3]. Many cases of early post-transplant cardiovascular complications are related to a high prevalence of comorbidity already present before transplantation, including hypertension, glucose intolerance, dyslipidemia and ischemic heart disease [4,5]. In addition, because of more liberal criteria of acceptance in the waiting list, the number of candidates with risk factors for CVD is increasing and many KT candidates continue to smoke, despite many health campaigns. Finally, several common risk factors, such as obesity, diabetes, hypertension and dyslipidemia, are worsened by the posttransplant effects of immunosuppressive regimens [6], and uncommon risk factors can develop after transplantation. Thus, a pretransplant cardiovascular risk scoring and an adequate correction of modifiable abnormalities are recommended during the evaluation and the preparation of a transplant candidate to reduce the risk of cardiovascular morbidity and mortality. In this review, we report the most important risk factors involved in post-transplant major cardiovascular events, keeping in mind that CVD is the result of interaction of genetic, environmental and acquired conditions (Figure 1).

## 2. Post-Transplant Diabetes Mellitus

Post-transplant diabetes mellitus (PTDM) develops in 20–30% of KT patients and represents a frequent cause of cardiovascular events and mortality [7,8]. PTDM includes all newly diagnosed diabetes mellitus (DM) in the post-transplant setting, irrespectively of the timing of diagnosis or whether DM was present but undetected before transplantation [9]. The diagnosis of DM must be performed according to American Diabetes Association guidelines [10]. Risk factors for PTDM are the same as type 2 DM: familial predisposition, age, overweight, dyslipidemia, hypertension etc. The likelihood of PTDM is higher in patients with risk factors for DM already present before transplantation [11]. However, a major role is also played by immunosuppressive drugs. Glucocorticoids exert diabetogenic effects with different mechanisms [12,13,14,15,16,17,18,19]. They increase appetite, promote leptin resistance and decrease glucose uptake in myocytes. In vitro studies demonstrated that glucocorticoids may alter the functions and induce apoptosis of β-cells. In the liver, they upregulate enzymes involved in gluconeogenesis and promote insulin resistance. In adipocytes, glucocorticoids decrease glucose uptake. Moreover, glucocorticoids suppress the expression of osteocalcin, which promotes insulin secretion by β-cells, thereby indirectly inhibiting insulin secretion [12,13]. Calcineurin inhibitors (CNI) are involved in the development of diabetes after KT. They impair insulin secretion and cause insulin resistance [14,15]. Tacrolimus is more diabetogenic than cyclosporine [16], probably because tacrolimus can exert a direct toxicity on pancreatic cells and can favor glucose reabsorption, as shown by experimental studies [17,18]. MammaBlagosklonny19lian target of rapamycin (mTOR) inhibitors can induce insulin sensitivity or insulin resistance. They have a mild diabetogenic impact in KT [19]. The prevention of PTDM and CVD includes reduced caloric intake and physical exercise to maintain a healthy body weight. Hypomagnesemia is an independent risk factor of PTDM, since magnesium regulates insulin secretion and sensitivity [20]. It is recommended to closely monitor the magnesium levels in the post-transplant period [21].

**Figure 2:**
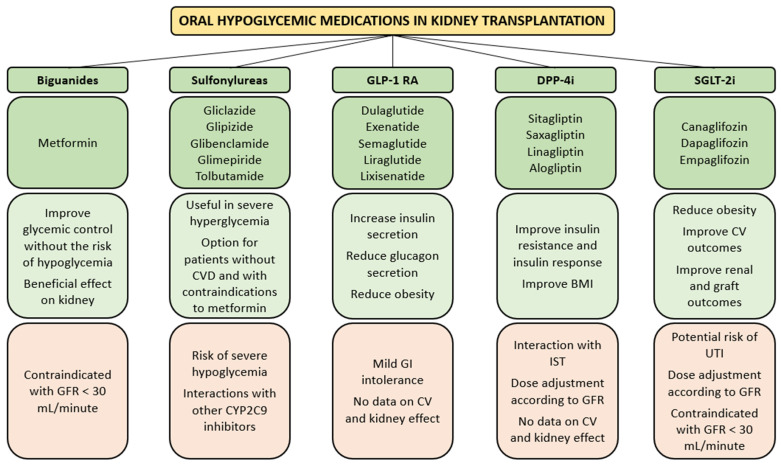
Most frequently used oral hypoglycemic medications (dark green) in kidney transplantation, with the main advantages (light green) and concerns (light orange). BMI, body mass index; CV, cardiovascular; CVD, cardiovascular disease; CYP2C9, cytochrome P450 family 2 subfamily C member 9; GFR, glomerular filtration rate; GI, gastrointestinal; DPP-4i, dipeptidyl peptidase-4 inhibitors GLP-1 RA, glucagon-like peptide-1 receptor agonists; IST, immunosuppressive therapy; SGLT-2i, sodium glucose cotransporter-2 inhibitors.

The management of diabetes after renal transplant is challenging. Current evidence suggests that metformin is the first-line treatment in patients without atherosclerotic cardiovascular disease, chronic kidney disease (CKD) and heart failure. However, if one or more of these comorbidities are present, a sodium glucose cotransporter-2 inhibitor (SGLT2i) or alternatively a glucagon-like peptide-1 receptor agonists (GLP-1 RA) should be started together with metformin [22]. In Figure 2, the main advantages and disadvantages of oral hypoglycemic drugs used in KT are reported.

Metformin has been shown to exert beneficial effects on the kidney in various clinical trials and experimental studies [23]. However, it is still contraindicated in patients with glomerular filtration rate (GFR) < 30 mL/minute [24]. Severe hypoglycemia may occur with sulfonylureas, because of the drug–drug interaction with azole antifungals or other inhibitors of Cytochrome P450 family 2 subfamily C member 9. [25]. SGLT2i reduces the reabsorption of glucose in the proximal tubular cells and facilitate its excretion in urine. As glucose is excreted, its plasma levels fall, leading to an improvement in all glycemic parameter. SGLT2 inhibition has been shown to reduce cardiovascular mortality and preserve kidney function in patients with type 2 DM [26,27]. Potential concerns of SGLT2 inhibition in KT recipients include volume depletion and urinary tract infections. A review of 9 studies, including 144 KT patients with diabetes, reported that SGTL2 inhibition resulted in a modest improvement in glycemic control, weight reduction, small reduction in blood pressure and stable renal allograft function. No serious side effects were reported [28]. In KT recipients, an improvement of glycemic control has been observed when SGLT2 inhibitors were added to other antidiabetic medications [29,30]. This class of drugs has not been tested yet in patients with severe reduction of GFR (<30 mL/minute) [31]. Additionally, dipeptidyl peptidase-4 inhibitors (DPP-4i) and GLP-1 RA are used in PTDM. GLP-1 RA and DPP-4i are incretin agents that stimulate β-cell function, decrease insulin resistance and slow gastric emptying [32]. Although data on GLP-1 RA in PTDM derive from small randomized controlled trials and case series, some benefits have been highlighted. GLP-1 RA are efficient in reducing glucagon secretion and increasing insulin secretion, thus improving glycemic control [33]. They favor weight loss through the delay of gastric emptying and the suppression of central appetite [34]. A reduction of cardiovascular events and a renoprotective effect have been demonstrated in type 2 DM patients treated with GLP-1 RA [32]. However, in KT recipients the same beneficial effect has not been described [32]. DPP-4i is effective in improving insulin resistance and improving glycemic control, as demonstrated by several studies [32]. DPP-4i can also favor body mass index (BMI) reduction [35]. However, DPP-4i seems to be less effective in reducing hemoglobin A1c compared to GLP-1 RA [36] No data are available on the effect of DPP-4i on major adverse cardiovascular events in KT recipients.

Despite oral hypoglycemic medications being preferred to insulin in the treatment of PTDM, about 50% of diabetic patients require insulin treatment. The dose and the type of insulin should be decided on an individual basis. It is safer to obtain acceptable glucose levels rather than insist on “ideal” lower glucose levels [11]. Attention should be paid to the Somogyi effect and the dawn phenomenon. It is, therefore, recommended to keep the values of glycemia in the early morning over 65 mg/dL.

## 3. Arterial Hypertension

Hypertension (HTN) is one of the strongest risk factors for CVD [37]. HTN is common after KT and ranges from about 50% to 80% in both adults and children [38,39,40]. However, a shared definition of post-kidney transplantation HTN does not exist and may be just clinically defined as a persistently elevated or a new onset elevation of blood pressure after successful KT [41]. In addition to poorer renal allograft outcomes, post-kidney transplantation HTN is associated with an increased risk of CVD. It has been demonstrated in the FAVORITE trial that higher systolic blood pressure is strongly and independently associated with increased risk of CVD and all-cause mortality in KT recipients [42]. Additionally, alterations of pulse pressure are associated with cardiovascular morbidity and mortality [43].

Several factors may cause or aggravate post-kidney transplantation HTN. Among these, immunosuppressive therapy with CNI and steroids has an important role. CNI may induce HTN through the alteration of vascular tone and interfering with renal sodium transport handling [41]. CNI-induced HTN is more evident with cyclosporine than tacrolimus [44,45,46]. Although the exact mechanism is not known, CNI may activate the sympathetic system and increase systemic vascular resistance by activating vasoconstrictor factors while reducing the production of vasodilator compounds, such as nitric oxide [47]. The sympathetic overactivity, together with a reduced GFR caused by the afferent arteriole constriction, determines an increased salt and water retention, with subsequent expansion of extracellular fluids [48]. Moreover, CNI may exalt the salt-sensitive component of HTN by activating the thiazide-sensitive sodium chloride cotransporter through an effect on the with-no-lysine [K] kinase (WNK) and SPS1-related proline/alanine-rich kinase (SPAK) [49]. Steroids can cause HTN through different mechanisms that eventually cause an increase of renal vascular resistance and the retention of water and salt. In fact, they exert mineralocorticoid effects, activate the renin-angiotensin system, potentiate vasoactive responses to catecholamines, and suppress the production of vasodilators, such as prostacyclin and nitric oxide [50]. Because of this effect on HTN, steroids avoidance or withdrawal during maintenance immunosuppressive treatment should be considered. A systematic review and meta-analysis showed that steroids avoidance or withdrawal is associated with better cardiovascular outcomes at a cost of an increased risk of acute rejections [51]. However, data on this approach are still conflicting. Another cause of HTN post-KT is renal artery stenosis (TRAS), which is a common vascular complication. It may also lead to allograft dysfunction. Its reported incidence ranges between 1% and 23% [52]. The pathogenesis of TRAS is related to vascular damage at the time of surgical anastomosis [53], but also to immune-mediated vascular endothelial injury [54]. The pharmacological treatment of TRAS is based on Renin-Angiotensin-Aldosterone System (RAAS) inhibition with angiotensin converting enzyme inhibitors (ACEi) and angiotensin receptor blockers (ARB). However, in the case of worsening renal function and/or uncontrolled HTN, renal artery angioplasty with stenting or surgical revascularization should be considered. There are no randomized controlled clinical trials comparing pharmacological treatment vs. renal artery angioplasty with stenting vs. surgical revascularization in TRAS, and data from non-transplant renal artery stenosis are also not conclusive [41]. Generally, surgical revascularization is considered in the case of unsuccessful renal artery angioplasty with stenting. Finally, other causes of post-transplant hypertension are renin production by failed native kidneys [55], weight gain after transplantation [56] and chronic renal allograft dysfunction [40].

Weight control, sodium restriction, low fat intake, physical exercise and cessation of smoking are strongly recommended to prevent hypertension. The target blood pressure in renal transplant recipients should be <130 mm Hg systolic and <80 mm Hg diastolic, regardless of other risk factors [57,58]. Calcium channel blockers, beta-blockers, diuretics, ACEi and ARB are the antihypertensive drugs that proved to reduce the risk of CVD in the general population [59]. These drugs are also frequently used in KT, with calcium channel blockers and beta-blockers as the most used first-line treatment. The characteristics of the most frequently used antihypertensive drugs in KT are reported in Table 1.

In the case of resistant HTN, defined as uncontrolled blood pressure despite a treatment with three antihypertensive drugs of which one is a diuretic, secondary forms of HTN must be ruled out. Apart from classical causes, renin production by failed native kidneys may be a cause of resistant HTN [55,68]. In this case, pharmacological treatment with RAAS inhibition or native nephrectomy should be considered. Several studies demonstrated that after native bilateral nephrectomy a better blood pressure control with a reduction of antihypertensive medications requirement is achieved both in adult and pediatric transplant recipients [69,70,71,72].

In addition to the RAAS, failed native kidneys are associated with an overactivity of the sympathetic nervous system [73]. Grounded on this pathogenic mechanism, native renal denervation (RDN) has been investigated in renal transplantation [41]. Complete RDN performed through bilateral native nephrectomy has been shown to improve HTN [72]. However, bilateral native nephrectomy may be burdened by surgical complications and should be considered for selected patients with severe and uncontrolled HTN. Another feasible approach is catheter-based RDN of native kidneys, although data are still scant. Two clinical case reports demonstrated an improvement of hypertension after RDN of native kidneys [74,75]. In a single center randomized controlled trial on 18 KT recipients with resistant HTN, a better control of blood pressure was achieved with RDN compared to pharmacological treatment alone [76].

The choice of the best treatment for post-kidney transplantation HTN should take into account the heterogeneity of the pathogenesis of HTN in this setting and the peculiar properties of antihypertensive medications, surgical nephrectomy and RDN. Considering all these factors, the clinician should tailor the treatment to the single patient.

## 4. Dyslipidemia

Lipid disorders are frequent in KT recipients and may contribute to cardiovascular morbidity and mortality both in adult and pediatric transplant recipients [39,77,78]. Advanced age, smoking, diabetes, obesity, excessive intake of calories and/or saturated fats, alcohol, high sugar beverages or foods, poor physical activity, hypothyroidism, proteinuria, renal insufficiency, use of beta-blockers or diuretics, and genetic factors can predispose to hypercholesterolemia and/or hypertriglyceridemia [79]. Immunosuppressive drugs can also contribute to dyslipidemia. Glucocorticoids may enhance the activity of acetyl-coenzyme A carboxylase and free fatty acid synthetase, increase hepatic synthesis of very low-density lipoproteins (VLDL), downregulate low-density lipoproteins (LDL) receptor activity of 3-hydroxy-3methylglutaryl coenzyme A (HMG-CoA) reductase and inhibit lipoprotein lipase [80]. While tacrolimus does not play a significant role in post-transplant hypercholesterolemia [81], cyclosporine downregulates the expression of the LDL receptor by increasing proprotein convertase subtilisin/kexin type 9 (PCSK9), an enzyme which degrades LDL receptors and decreases the transport of cholesterol to the bowel through the inhibition of cholesterol 7-alpha-hydroxylase [82,83]. Hypertriglyceridemia can occur in tacrolimus-treated patients and may be favored by high levels of the drug and decrease of lipoprotein lipase activity [84]. The mTOR inhibitors can frequently induce hypercholesterolemia. The mechanisms are still poorly elucidated. It is likely that mTOR inhibitors increase PCSK9 levels which are strong regulators of LDL cholesterol levels [85]; they can also increase adipose tissue lipase activity and/or decrease lipoprotein lipase activity [86]. On the other hand, both sirolimus and everolimus may exert cardioprotective effects, by inhibiting intimal proliferation and stabilizing the atherosclerotic plaque [87].

**Figure 3:**
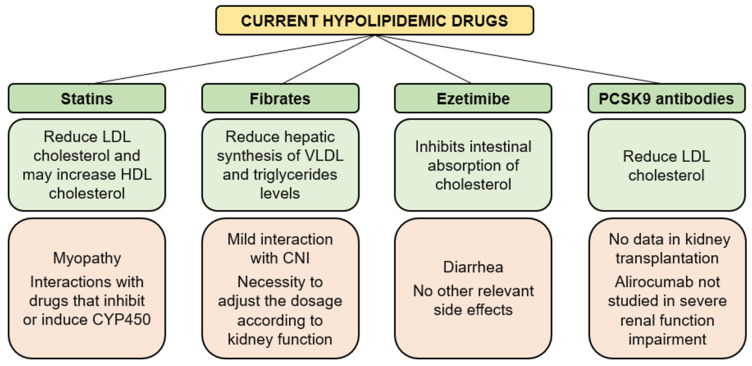
Advantages (light green) and concerns (light orange) of the available hypolipidemic drugs. CNI, calcineurin inhibitors; CYP450, cytochromes P450; HDL, high-density lipoproteins; LDL, low-density lipoproteins; PCSK9, proprotein convertase subtilisin/kexin type 9.

The management of dyslipidemia includes physical activity, diet (poor intake of saturated fatty acids, particularly animal fats) and reduced doses of immunosuppressive agents and lipid-lowering agents (Figure 3). Patients with hypertriglyceridemia should be placed on a hypocaloric diet that restricts the intake of simple sugars and alcohol, while limiting fat intake to less than 30% of total daily calories.

HMG-CoA reductase inhibitors, also called statins, may reduce LDL, increase high-density lipoproteins (HDL) and slightly decrease triglycerides [88]. Statins are the first-line treatment of hypercholesterolemia and are recommended for people with an LDL cholesterol level higher than 190 mg/dL [89]. Since statins and CNI are both metabolized by the P450 cytochrome (CYP450) enzymatic system, pharmacokinetic interactions are likely to occur. A Cox regression analysis on 622 KT recipients followed in mean for 5.4 years showed that statins did not significantly decrease incident cardiovascular events, possibly due to a bilateral pharmacological interaction [90]. Statins are generally well tolerated, but myopathy may occur. Statin-associated myopathy, with significant elevation of serum creatine kinase (CK), is a rare but serious side effect of statins, affecting 1 per 1000 to 1 per 10,000 people on standard statin doses.

Fibrates (clofibrate, bezafibrate, fenofibrate, ciprofibrate and gemfibrozil) induce a reduction in hepatic VLDL synthesis and an increase in lipoprotein lipase activity, thereby causing a reduction in triglycerides concentrations and an increase in HDL cholesterol concentration. A systematic review and meta-analysis demonstrated that fibrates could improve lipid profiles and prevent cardiovascular events in people with CKD. Fibrates can also reduce albuminuria and reversibly increase serum creatinine [91]. Probably, there is little interaction between fibrates and CNI, but to be on the safe side, in transplant patients, it is better not to exceed a daily dose of 900 mg for gemfibrozil, 400 mg for bezafibrate or 200 mg for fenofibrate.

Ezetimibe selectively inhibits the intestinal absorption of cholesterol and related phytosterols. At a dose of 10 mg/day, ezetimibe can reduce hypercholesterolemia and hypertriglyceridemia in renal transplant recipient given maximal doses of statin [92]. In transplant patients resistant to statins, the addition of ezetimibe determined a decrease of total cholesterol, LDL and triglycerides by 21%, 31% and 13%, respectively. CK, liver enzyme serum levels and renal function were not affected to any level of clinical significance with the addition of ezetimibe [93].

Anti-PCSK9 antibodies, alirocumab and evolocumab are monoclonal antibodies approved for clinical use in the treatment of hypercholesterolemia. They have a significant activity in lowering cholesterol levels and reducing the risk for CVD, without relevant safety issues [94]. Angiopoietin-like protein3 and apolipoprotein C-III are regulators of triglycerides that have been identified as targeting agents in recent clinical trials [95]. However, no data are available about the use of these new drugs in KT.

## 5. Obesity

The percentage of obese patients has steadily increased in recent decades, and since obesity is a risk factor for end-stage renal disease, the same increase has been also observed in renal transplant candidates [96]. Obesity is not only associated with an increased risk of surgery complications [97], but also negatively influences graft survival and determines a higher risk of CVD [96]. A systematic review found that a BMI >30 kg/m^2^ was associated with increased mortality and decreased graft survival compared to patients with a BMI < 30 kg/m^2^ [98].

It has been demonstrated that a 5 unit increase in recipient BMI can increase the risk of cardiac disease by 25% [99]. However, it is very difficult to determine the real impact of obesity on CVD, since it is a well-recognized risk factor for hypertension, dyslipidemia, impaired glucose tolerance and proteinuria, all of which increase cardiovascular risk [100]. Probably for this reason, systematic reviews did not find a statistically significant association between obesity and cardiovascular mortality [101]. However, considering the effect of obesity on other risk factors, weight loss through dietary advice and physical activity should be encouraged in all renal transplant recipients with BMI > 30 kg/m^2^.

## 6. Graft Dysfunction and Proteinuria

Many events may be responsible of progressive graft dysfunction, including rejection, recurrent or de novo renal and systemic diseases, drug-related nephrotoxicity, polyoma BK virus nephropathy, poor quality of the donated kidney, poor adherence to prescriptions, etc. Whatever the cause, in KT, a low estimated GFR (eGFR) is a major risk factor for cardiovascular events [102]. The association of graft dysfunction with conventional risk factors, such as arterial hypertension, glucose intolerance and abnormal lipoprotein, and other risk factors, such as anemia, oxidative stress and inflammation, may contribute to the development of CVD in patients with reduced kidney graft function. Persistent proteinuria is an independent risk factor for increased cardiovascular morbidity and mortality in renal transplant patients [103]. The impact of proteinuria on CVD is even greater when it is associated with poor renal allograft function, suggesting that eGFR and albuminuria should be used together to determine the risk of cardiovascular outcomes in transplant recipients [104].

## 7. Hyperuricemia

In KT, the causal relationship between hyperuricemia and CVD remains debated [105]. However, uric acid may be pathogenic and participate in the pathophysiology of CVD by serving as a bridging mechanism that mediates or potentiates the deleterious effects of cardiovascular risk factors on vascular tissue and myocardium [106]. Allopurinol remains the cornerstone drug to reduce uricemia to ≤ 6 mg/dL, which is considered the minimum target of urate-lowering drugs [107]. Febuxostat is an acceptable alternative for the treatment of hyperuricemia. A prospective, randomized trial of febuxostat versus allopurinol showed that the two drugs were comparable with respect to the primary cardiovascular endpoint and long-terms untoward events [108]. Additionally, a retrospective study comparing febuxostat and allopurinol in early post-renal transplant recipients did not find differences in the efficacy and safety between the two drugs [109]. Febuxostat dose adjustment is not necessary in patients with renal function impairment [110]. Both allopurinol and febuxostat inhibit the activity of xanthine oxidase, an enzyme involved in the metabolism of 6-mercaptopurine. Caution should be taken before administering these drugs to patients taking azathioprine. To prevent bone marrow aplasia, it is mandatory to reduce the dose of azathioprine to one third and to check blood count frequently.

## 8. Hyperhomocysteinemia

Elevated levels of homocysteine are common in renal transplant recipients and are mainly associated with graft function and only to a lesser degree with vitamin status [110]. Whether hyperhomocysteinemia in renal transplant recipients is an innocent marker of renal dysfunction or an important risk factor for atherosclerosis is still discussed. Retrospective studies concluded that plasma levels of homocysteine are neither correlated with patient nor with graft survival [111], while prospective studies demonstrated that they represent an independent variable associated with cardiovascular events [112]. In a large randomized controlled trial, 4110 stable KT recipients were assigned to a high dose or low dose of multivitamin therapy, which included folic acid and vitamins B6 and B12. Treatment with a high-dose multivitamin preparation did not reduce a composite CVD outcome or all-cause mortality, despite significant reduction in homocysteine level [113]. A large review of the literature also concluded that there is no evidence to support the use of homocysteine-lowering therapy for cardiovascular disease prevention in KT recipients [114]. At present, treatments with folic acid and vitamins B6 and B12 do not represent a therapeutic solution for preventing CVD after KT.

## 9. Inflammation and Oxidative Stress

Inflammation and oxidative stress are known risk factors for CVD in KT recipients [115]. Several factors, such as donor brain death, ischemia-reperfusion injury, rejection, infection and chronic allograft dysfunction, may induce an inflammatory state in KT. Furthermore, inflammatory cells, cytokines, growth factors, complement, coagulation cascade and oxidative stress create an unbalanced interaction with innate and adaptive immunity, which are both heavily involved in atherogenesis [116,117]. In addition, a prothrombotic state and reduced fibrinolysis are present in KT. The interactions between inflammation and thrombosis may increase the risk of CVD [118]. Inflammation is also associated with elevated levels of fibroblast growth factor 23 (FGF23) and low levels of Klotho, which contribute to major adverse cardiovascular events [119,120]. Another major consequence of the inflammatory/oxidative state is the development of chronic hypoxia [121]. Through the mediation of interleukins 1 and 6, angiotensin II and transforming growth factor-β, hypoxia can result in excessive accumulation of extracellular matrix [122,123]. The final result of extracellular matrix (ECM) deposition is the development of tubulointerstitial fibrosis and vascular ischemia, which can further aggravate hypoxia and activate the innate immune system, leading to uncontrolled intra-graft fibrogenesis and allograft dysfunction, disruption and replacement of functional parenchyma, release of inflammatory mediators and reactive oxygen species and induction of epithelial–mesenchymal transition [124]. Oxidative stress increase is directly correlated to the reduction of graft function [125]. Lifestyle and regular physical activity may reduce inflammation and oxidative stress. No prospective controlled trial to assess the validity of different drugs has been conducted in KT.

## 10. Klotho and Fibroblast Growth Factor 23

Klotho is a transmembrane protein produced by kidney, which is cleaved by secretases and released into the circulation as soluble Klotho, which functions as a coreceptor of Fibroblast Growing Factor (FGF) receptors to activate FGF23. There is evidence that Klotho deficiency is a pathogenic factor for CVD and excess of FGF23 may also contribute to CVD [119,126]. Most candidates to renal transplant present low levels of Klotho and elevated levels of FGF23 [127,128]. The level of Klotho tends to decrease in the presence of kidney graft insufficiency with a correspondent increase in levels of FGF23 [129]. A study in KT recipients demonstrated that recombinant human erythropoietin may mitigate the Klotho reduction caused by renal damage [130].

## 11. Vascular Calcifications

Vascular calcifications are frequent in KT recipients and are associated with an increased risk of CVD and mortality [131]. In many cases, calcifications are already present before transplantation and may be caused by hypocalcemia, hyperphosphatemia, secondary hyperparathyroidism, decreased vitamin D and/or increased levels of osteoprogeterin and sclerostin [132]. After transplantation, some of these disorders may improve, but low-grade inflammation with persistent redox imbalance and deregulated mineral and bone metabolism can accelerate vascular calcifications [133]. A further contributor to vascular calcifications is osteoprotegerin cytokine receptor of the tumor necrosis factor receptor superfamily, which can cause arterial stiffness in KT recipients [134]. The calcification propensity is associated with an increased risk of all-cause mortality and cardiovascular mortality in KT recipients [135].

Prevention of vascular calcifications rests on lowering the circulating levels of both phosphate and calcium. Current treatments are based on calcimimetics, bisphosphonates, thiosulfate, and supplement of vitamin D in case of deficiency [136]. In patients with vascular calcifications vitamin K deficiency is frequent [137], but a double-blind, placebo-controlled trial reported that vitamin K supplementation did not reduce vascular stiffness or calcification in KT recipients [137]. Pyrophosphate, an endogenous inhibitor of calcification, proved to prevent calcifications in animal studies, but has not been investigated in KT recipients.

## 12. Smoking

Smoking represents one of the most important preventable risk factors for the development of cardiovascular events. In organ transplant recipients, tobacco smoking is associated with CVD and decreased patient and graft survival [138]. In a series of 864 adult renal transplant recipients, smoking increased the risk of CVD by 30% [139]. In a retrospective cohort of 41,705 adult renal transplant recipients, incident smoking after transplant was associated with a risk of death more than doubled in comparison with never smokers [140]. Smoking cessation may be associated with substantial health benefits for all smokers [141]. Since the successful rate of smoking cessation without a treatment is poor, smoking cessation programs are emerging as an important part of KT recipients’ treatment [142]. When counseling alone is not sufficient, a pharmacological treatment should be considered. Nicotine replacement therapy (NRT) is effective and has no contraindications and few adverse effects [143]. Bupronion is an antidepressant which inhibits dopamine and norepinephrine uptake. It is as effective as NRT, but is burdened by several adverse effects (e.g., rash, headache, dry mouth, dizziness and sleep disorder), and requires dose adjustment in advanced stages of CKD [144]. Finally, two nicotinic cholinergic receptor partial agonists, varenicline and cytisine, are effective for smoking cessation with mild adverse effects (nausea, vomiting and sleep disorders) [142].

## 13. Physical Inactivity

Poor physical activity at the time of KT is a strong predictor of all-cause mortality, particularly in older recipients, smokers and diabetics [145]. However, most transplant recipients, in particular elderly subjects and children, are sedentary. Many patients do not exert physical activity because they believe that their disability on dialysis did not change following transplantation. In a study on renal transplant recipients, the risk of cardiovascular death and all-cause mortality was significantly higher in patients with poor physical activity [146]. In a large multiethnic, multicenter trial on homocysteine in KT recipients, lower levels of physical activity were associated with more CVD risk factors [145]. In transplanted children, a weekly physical exercise of 3–5 h significantly improved cardiorespiratory fitness and the left ventricular mass [147]. To reduce the burden of cardiovascular risk factors in KT recipients, prehabilitation before KT and post-transplant physical activity should be encouraged [148,149,150].

## 14. Anemia

Anemia is common after KT [151]. Late anemia (>6 months) is mainly caused by impaired kidney graft function and defective erythropoietin production. Iron deficiency is common and is associated with an increased mortality risk in KT recipients [152]. ACEi, azathioprine, mycophenolate salts, mTOR inhibitors and viral infections may trigger or aggravate anemia.

Chronic anemia may increase preload, reduce afterload and lead to a hyperdynamic state with increased cardiac output, left ventricular hypertrophy and maladaptive left ventricular hypertrophy, which in turn is a well-recognized risk factor for CVD outcomes and all-cause mortality [153]. Anemia has been reported to be an independent risk factor for de novo congestive heart failure in renal transplant recipients [154]. Erythropoiesis-stimulating agents and iron supplementation are currently used to correct post-transplant anemia

## 15. Vitamin D Deficiency

Vitamin D is metabolized first to 25-hydroxyvitamin D [25(OH)D] and is hydrolyzed in the kidney to the active form 1,25(OH)D_2_ or vitamin D3. Vitamin D regulates renin-angiotensin-aldosterone system, nitric oxide production, and exert anti-oxidant and anti-inflammatory effects [155]. The recommended 25(OH)D level after transplant is at least 30 ng/mL (75 nmol/L). Vitamin D deficiency is frequent after KT [156] and this condition may render transplant recipients more susceptible to cardiovascular events and mortality [157]. Despite this, many trials have failed to find a beneficial effect of vitamin D supplements on CVD outcomes and there are some concerns related to calcium use and increased CVD risk [158]. These findings, along with the lack of consensus on optimal serum 25(OH)D concentrations, have reduced the initial enthusiasm for vitamin D supplements.

## 16. Quality of Life

Although Quality of Life (QoL) scores tend to improve after transplantation [159], KT recipients experience significant physical and psychosocial challenges and several difficulties, including health anxiety, body image concerns, sleep disorders and depressive symptoms [160,161]. Little is known about the association between QoL and cardiovascular risk factors in KT. However, in the general population, psychological factors, such as major depression and stress, are now known as independent risk factors for developing CVD [162,163]. Interventions such as increasing exercise and training appear to be safe in KT recipients and are associated with improved QoL and exercise capacity [149,164]. Sleeplessness may be reduced by avoiding or reducing the use of caffeine, alcohol and medications that can cause insomnia. Administering short-acting glucocorticoids in a single morning administration may reduce sleeplessness.

## 17. Sleep Apnea

In the general population, sleep apnea (SA) increases the risk of CVD independently from other cardiovascular risk factors [165]. SA prevalence in CKD patients is about 27% and increases in parallel with the reduction of GFR, with a prevalence of 57% in patients with end-stage kidney disease [166]. The higher prevalence seems to be correlated to fluid overload, uremic toxicity and altered chemosensitivity, but this hypothesis needs to be confirmed [167]. Although the improvement of both fluid overload and uremic toxicity after KT, SA recurs on long term observation. However, in this population, SA is not associated with poorer renal allograft outcomes or increased CVD [167].

## 18. Conclusions

Cardiovascular disease is a frequent cause of morbidity after KT and represents the leading cause of mortality [1]. The pathogenesis of cardiovascular disease in KT recipients is multifactorial, as has been described in this review. Modifiable risk factors that develop after KT represent possible therapeutic targets to prevent CVD. After transplantation, smoking cessation, maintenance of ideal body weight, healthy diet, physical activity, and adherence to prescriptions are strongly recommended. Whenever possible, the immunosuppression should be tailored to the clinical characteristics of the patient, by reducing the use of glucocorticoids, CNI or other drugs in patients with major risk factors for CVD. Treatment of post-transplant diabetes, hypertension and/or hyperlipidemia should be timely and vigorous. Prevention and treatment of anemia is also important. Still under discussion are other measures to prevent CVD, including urate-lowering therapy, supplementation of vitamin D, treatment with folic acid, vitamin B6 and B12 and use of anti-inflammatory and anti-oxidant drugs. Reduction of cardiovascular morbidity can improve not only the life expectancy and the quality of life of the transplant recipients, but also their allograft function and survival.

## Figures and Tables

**Table 1:** Main advantages and side effects of the drugs used in the treatment of hypertension in renal transplant recipients.

Antihypertensive Classes	References	Advantages	Side Effects
Thiazide Diuretics	Taber D. et al. [60]	Reduce extracellular expansion Reduce arteriolar resistance Consider in salt-sensitive HTN	Hypokalemia, hyponatremia Reversible increase in serum creatinine
Loop Diuretics	Rizk J. et al. [61]	Reduce extracellular expansion Effective in heart failure	Hypokalemia, hyponatremia, hypomagnesemia Hypovolemia Ototoxicity
Calcium Channel Blockers	Baroletti S. et al. [62]	Reduce arteriolar vasoconstriction Reverse ventricular hypertrophy	Peripheral oedema Gastroesophageal reflux Gum hypertrophy Non-dihydropyridine calcium channel blockers increase cyclosporine levels
RAAS-inhibitors (ACEi and ARB)	Jiang Y. et al. [63]	Prevent heart failure Prevent intimal thickening Antiproteinuric effects	Small increase in serum creatinine Hyperkalemia Anemia Worsening renal function in the setting of TRAS or hypovolemia
Mineralcorticoid Receptor Antagonists	Girerd S. et al. [64]	Improve outcomes in HFrEF	Hyperkalemia
Beta-blockers	Aftab W. et al. [65]	Cardioprotective	Hyperlipidemia Interference with glucose metabolism Hypoglycemia in diabetic patients
Alpha_2_ adrenergic agonists	Gavras I. et al. [66]	Peripheral vasodilation No change in renal plasma flow and GFR	Potential rebound HTN Orthostatic hypotension Dryness Confusion Constipation
Alpha_1_ antagonists	Martinez-Castelao A. et al. [67]	Peripheral vasodilation	Headache, drowsiness, numbness Constipation

ACEi, angiotensin converting enzyme inhibitors; ARB, angiotensin receptor blockers; GFR, glomerular filtration rate; HFrEF, heart failure with reduced ejection fraction; HTN, hypertension; RAAS, Renin-Angiotensin-Aldosterone System; TRAS, transplant renal artery stenosis.

## Data Availability

Not applicable.

## Abbreviations

25(OH)D	25-hydroxyvitamin D
ACE	Angiotensin converting enzyme
ACEi	Angiotensin converting enzyme inhibitors
apoCIII	Apolipoprotein CIII
ARB	Angiotensin receptor blockers
BMI	Body mass index
CK	Creatin kinase
CKD	Chronic kidney disease
CNI	Calcineurin inhibitors
CVD	Cardiovascular disease
CYP	Cytochromes P
CYP2C9	Cytochrome P450 family 2 subfamily C member 9
CYP450	P450 cytochrome
DPP-4i	Dipeptidyl peptidase-4 inhibitors
ECM	Extracellular matrix
eGFR	Estimated glomerular filtration rate
FGF	Fibroblast growing factors
FGF23	Fibroblast growth factor23
GI	Gastrointestinal
GFR	Glomerular filtration rate
GLP-1 RA	Glucagon-like peptide-1 receptor agonists
HFrEF	Heart failure with reduced ejection fraction
HDL	High-density lipoprotein
HMG-CoA	3-hydroxy-3-methyl-glutaryl-coenzyme A
HTN	Hypertension
IST	Immunosuppressive therapy
KT	Kidney transplantation
LDL	Low-density lipoprotein
mTOR	Mammalian target of rapamycin
NRT	Nicotine replacement therapy
PCSK9	Proprotein convertase subtilisin/kexin type 9
PTDM	Post-transplant diabetes mellitus
QoL	Quality of Life
RAAS	Renin-Angiotensin-Aldosterone System
RDN	Renal denervation
SA	Sleep apnea
SGLT2i	Sodium glucose cotransporter-2 inhibitors
SPAK	SPS1-related proline/alanine-rich kinase
TRAS	Transplant renal artery stenosis
VLDL	Very low-density lipoprotein
WNK	With-no-lysine [K] kinase
